# Positive association of serum FUT8 activity with renal tubulointerstitial injury in IgA nephropathy patients

**DOI:** 10.1002/iid3.686

**Published:** 2022-08-29

**Authors:** Ning Yang, Long‐kai Li, Hui He, Xia‐nan Guo, Xue‐feng Yuan, Zhi‐tong Li, Wei‐dong Wang, Biao‐jie Qin, Xiang‐ning Du, Xu Zhang, Shu‐ni Chen, Hong‐li Lin

**Affiliations:** ^1^ Graduate School of Dalian Medical University Dalian Medical University Dalian China; ^2^ Department of Nephrology, Liaoning Translational Medicine Center of Nephrology The First Affiliated Hospital of Dalian Medical University Dalian China

**Keywords:** IgA nephropathy, serum FUT8 activity, tubulointerstitial injury

## Abstract

**Background:**

α‐1,6 Fucosyltransferase (FUT8) appears to play an essential role in the pathogenesis of renal fibrosis. However, it remained unknown whether FUT8 also contributed to renal fibrosis in immunoglobulin A nephropathy (IgAN). In the present study, we explored the association of serum FUT8 activity with renal tubulointerstitial injury in IgAN patients.

**Methods:**

Serum FUT8 activity was measured in 135 IgAN patients and 68 healthy controls from January 2016 to December 2018. The relationships of serum FUT8 activity with clinical and pathological features were analyzed.

**Results:**

Relative to healthy controls, IgAN patients had significantly higher serum FUT8 activity and upregulation of renal FUT8 protein (*p* < .05). Among IgAN patients, there was a positive correlation of serum FUT8 activity with renal FUT8 protein expression (*p* < .05). Multivariable logistic regression analyses showed that serum FUT8 activity was significantly associated with serum creatinine and eGFR (*p* < .05). Based on a cut‐off value determined from ROC curve analysis, we divided IgAN patients into a low serum FUT8 activity group (≤12.2 pmol/h/mL, *n* = 40) and a high serum FUT8 activity group (>12.2 pmol/h/ml, *n* = 95). The high serum FUT8 activity group had a higher Oxford T score, increased inflammatory cell infiltration, more severe fibrosis and poor renal function (*p* < .05).

**Conclusion:**

Serum FUT8 activity was positive association with renal tubulointerstitial injury in IgAN patients.

## Graphical abstract

FUT8 appears to play an essential role in the pathogenesis of renal fibrosis. However, it remained unknown whether FUT8 also contributed to renal fibrosis in IgAN. In the present study, we explored the association of serum FUT8 activity with renal tubulointerstitial injury in IgAN patients.

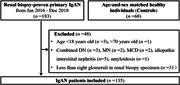

## 1 INTRODUCTION

Immunoglobulin A nephropathy (IgAN) is the most common primary glomerulonephritis worldwide.^1^ IgAN is a highly heterogeneous disease, with frequent involvement of tubulointerstitial injury. The severity of tubulointerstitial lesions in IgAN closely correlates with renal progression.^2^ The T score in the Oxford classification for tubulointerstitial injury in IgAN patients is the most reliable predictor of renal outcome.^3^ However, studies have found that interstitial lymphocyte and macrophage infiltration are also closely related to the prognosis of IgAN. In advanced IgAN, the positive proportion of CD3 + T lymphocyte infiltration was higher. The prognosis of IgAN patients with more macrophage infiltration is worse.^4^, ^5^ T score evaluation for renal interstitial injury ignores the evaluation of inflammation, and maybe not enough. In addition, T grading depends on the pathology of renal biopsy, which is an invasive operation and cannot be repeated. Therefore, an ideal noninvasive biomarker is needed to evaluate the tubulointerstitial injury in IgAN patients.

Core fucosylation, which is mediated by α‐1,6 fucosyltransferase (FUT8), is an important posttranslational modification.^6^ FUT8 is a unique enzyme that is responsible for core fucosylation in mammals.^7^ FUT8 promotes the transfer of a fucose residue from GDP‐fucose to the *N*‐acetylglucosamine (GlcNAc) residue of N‐linked glycans.^8^ Our previous animal studies found that FUT8 was markedly upregulated, it activated multiple fibrogenic signaling pathways, and its knockdown ameliorated the progression of renal and peritoneal fibrosis. Therefore, FUT8 appears to play an essential role in the pathogenesis of renal fibrosis.

Considering the role of FUT8 in renal fibrosis, we hypothesized that FUT8 may be associated with the renal tubulointerstitial injury in IgAN patients. In the present study, we measured serum FUT8 activity and investigated its association with renal tubulointerstitial injury in IgAN patients. Our study is the first to report elevated serum FUT8 enzyme activity in IgAN patients, which is significantly associated with renal interstitial injury. Serum FUT8 is expected to be an ideal biological marker for the evaluation of IgAN renal interstitial injury.

## 2 METHODS

### 2.1 Sample collection

**Figure 1:**
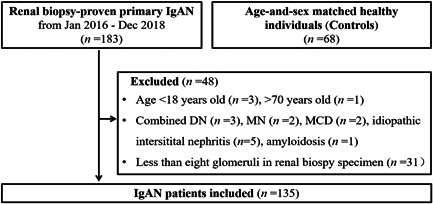
The flow of selection in our study. IgAN, immunoglobulin A nephropathy.

A total of 183 patients who were diagnosed with biopsy‐proven primary IgAN at the Nephrology Department of Dalian Medical University from January 2016 to December 2018 were initially examined. Each patient was 18–70 years old and had an eGFR greater than 30 ml/min/1.73 m^2^. The exclusion criteria were secondary IgAN or IgAN combined with other kidney diseases, acute interstitial nephritis, receipt of corticosteroid or immunosuppressant before renal biopsy, and fewer than 8 glomeruli collected from the renal biopsy. At the same time, sex‐and age‐matched healthy controls were recruited. The flow of selection was shown in Figure 1. Normal kidney tissues from six patients who received nephrectomy due to tumor were provided by the Urology Department of the First Affiliated Hospital of Dalian Medical University Medical University (Approval number: PJ‐ KY‐2016‐76). Each participant provided written informed consent before participation. The protocol was approved by the Institutional Ethical Committee of the First Affiliated Hospital of Dalian Medical University (Approval number: LCKY2013‐14).

### 2.2 Blood and urinary samples

Blood and urine samples were collected before the renal biopsy and were centrifuged at 4°C for 10 min at 3000 *g*. The supernatants were then immediately stored at −80°C before analysis.

### 2.3 Data collection

Sex, age, mean arterial pressure (MAP), serum albumin, cholesterol, hemoglobin, estimated glomerular filtration rate (eGFR), serum creatinine (Scr), blood urea nitrogen, uric acid, urinary protein, and urinary erythrocyte count were recorded at the time of renal biopsy. eGFR was calculated using the chronic kidney disease epidemiology equation (CKD‐EPI). All measurements were performed according to the manufacturer's protocols.

### 2.4 Assessment of renal histopathology

Two renal pathologists who were blinded to the clinical data evaluated the histological findings using the revised MEST‐C score in the Oxford IgAN classification, and recorded the percentages of inflammation cell infiltration, and vascular lesions, as previously reported.^9^, ^10^ The interstitial inflammation index (Inf I) was used to assess the extent of interstitial inflammatory cell infiltration (0: 0%; 1: <10%; 2: 10%–24%; 3: 25%–49%; 4: >50%). The vascular chronic index (VCI) was used to assess arterial hyaline change and vascular wall thickening (0: absent, 1: present).^10^ CD4^+^ T cells, CD8^+^ T cells, CD20^+^ B cells, and CD68^+^ macrophages were counted in five representative high‐power fields (HPFs, Cell Quant, 3DHISTECH) and the average number of cells per HPF was calculated.^11^

### 2.5 Serum and urine FUT8 activity

Serum and urine FUT8 activity were measured using HPLC, as previously described.^12^, ^13^ A serum or urine sample was added to a mixture of 2‐Morpholinoethanesulfonic acid (MES, pH 6.4), GDP‐l‐fucose, and GnGn‐Asn‐4‐(2‐pyridylamine) butylamine (PABA), incubated at 37°C for 12 h, heated at 100°C for 3 min, and then ddH_2_O was added. Finally, the mixture was centrifuged at 12,000 *g* for 10 min. The reaction products were identified by fluorescence using HPLC (Waters Corporation), with excitation at 320nm and emission at 400 nm. The ratio of urine FUT8 activity to urine creatinine concentration (mmol/L) was calculated.

### 2.6 Immunohistochemistry and immunofluorescence

The infiltration of T cells, B cells, and macrophages and the expression of TNF‐α, MCP‐1, FUT8, and α‐SMA were determined. CD4 (ab133616, Abcam), CD20 (ab78237, Abcam), CD8 (ZA‐0508, Maxim‐Bio), CD68 (Kit‐0026, Maxim‐Bio), MCP‐1 (419875, CST), TNF‐α (41504, SAB), and FUT8 (ab204124, Abcam) were measured using immunohistochemistry. α‐SMA (ab5694, Abcam) and LCA (B‐1045/RL‐1042 Vector Labs) were measured using immunofluorescence. Quantitation of these data was performed using Cell Quant software (3DHISTECH) and ImageJ version 1.8.0.

### 2.7 Western blot analysis

Serum samples were incubated with an anti‐FUT8 antibody (ab204124, Abcam), and then with an HRP‐conjugated secondary antibody (1:10,000, Zhongshan). Target proteins were visualized by electro‐chemiluminescence (ECL, Fuji film Corporation). Coomassie brilliant blue staining was performed on another gel to assure uniform loading.

### 2.8 Statistical analysis

Data were analyzed using SPSS version 25.0 (IBM, Armonk). Data are expressed as means ± standard deviations (normal distributions) or as medians and interquartile ranges (non‐normal distributions). Variables were compared using Student's *t‐*test or the Mann–Whitney *U* test, as appropriate. The relationships of serum FUT8 activity with clinical parameters were determined using Spearman's correlation analysis. Multivariable logistic regression was to determine the associations between serum FUT8 activity and serum creatinine, eGFR. The selection of covariates was based on factor showing a relation to serum FUT8 or renal function. The optimal cutoff value for serum FUT8 activity (low *vs*. high) was obtained by receiver operating characteristic (ROC) curve analysis and calculation of Youden's index. The differences in renal pathology of IgAN patients with low and high serum FUT8 activity were compared using the *χ* ^2^ test. A difference was considered significant if the *p* value was below .05.

## 3 RESULTS

### 3.1 Characteristics of IgAN patients and healthy controls

We enrolled 135 patients with biopsy‐proven primary IgAN, 68 healthy individuals as controls shown in Table 1. The IgAN patients had significantly higher mean arterial pressure (MAP), Scr, and blood urea nitrogen and significantly lower serum albumin and eGFR (all *p* < .05). There were no differences in hemoglobin, Chol, UA between IgAN patients and healthy individuals (all *p* > .05).

**Table 1:** Characteristics of IgAN patients and healthy controls

Item	Controls (*n* = 68)	IgAN patients (*n* = 135)	*p*
Male [*n* (%)]	31 (45.6%)	55 (40.7%)	NS
Age (years)	36.5 (26.0, 50.0)	36.0 (29.0, 48.0)	NS
Mean arterial pressure (mmHg)	88.0 (78.0, 97.0)	105 (100, 115)	**<.05**
Albumin (g/L)	44.9 (42.9, 46.0)	40.1 (36.0, 42.3)	**<.05**
Cholesterol (mmol/L)	4.7 ± 0.7	4.9 ± 1.1	NS
Hemoglobin (g/L)	133.5 (128.3, 139.8)	132.0 (123.0, 143.0)	NS
eGFR (mL/min/1.73 m^2^)	101.0 (92.5, 118.7)	88.4 (66.9, 110.3)	**<.05**
Serum creatinine (μmol/L)	57.5 (53.0, 61.8)	81.0 (62.0, 102.0)	**<.05**
Blood urea nitrogen (mmol/L)	4.4 (3.6, 5.0)	5.8 (4.8, 7.3)	**<.05**
Uric acid (mmol/L)	353.5 (321.3, 378.8)	380.0 (300.0, 441.1)	NS
Urine protein (mg/24 h)	—	1151.0 (486.0, 2150.0)	—
Microscopic hematuria [*n* (%)]	—	125 (92.6%)	—

*Note*: Values are expressed as means ± standard deviations or medians (25th, 75th percentiles).

Abbrevations: eGFR, estimated glomerular filtration rate; IgAN, immunoglobulin A nephropathy; NS, no significance.

### 3.2 IgAN patients had higher serum FUT8 activity and FUT8 protein

**Figure 2:**
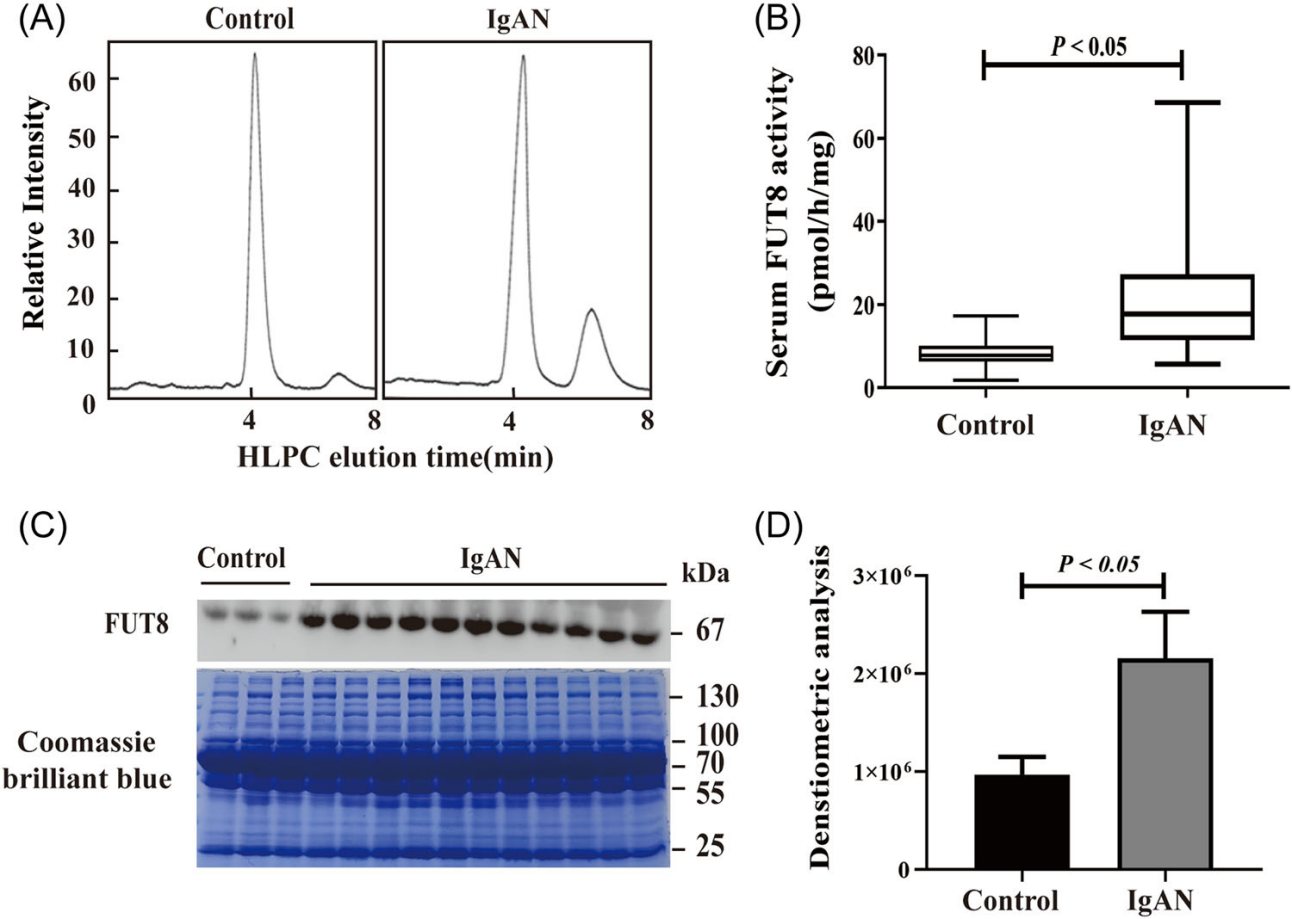
IgAN patients had higher serum FUT8 activity and serum FUT8 protein. (A) Representative HPLC results of serum FUT8 activity in a control patient and an IgAN patient. (B) Quantitative analysis of serum FUT8 activity in all controls and IgAN patients. (C) Representative western blot analysis of serum FUT8 protein in a control patientand a IgAN patient. (D) Quantitative analysis of serum FUT8 protein in all controls and IgAN patients. FUT8, α‐1,6 fucosyltransferase; HPLC, high‐performance liquid chromatography; IgAN, immunoglobulin A nephropathy

To investigate the possible role of FUT8 in IgAN, we measured serum FUT8 activity using HPLC and the serum FUT8 protein using western blot analysis. HPLC results showed that relative to the healthy controls, IgAN patients had a higher serum FUT8 activity (Figure 2A,B, *p* < .05). Serum samples from healthy controls and IgAN patients were stained to ensure the same amount of samples in each group. Western blot analysis found that serum FUT8 protein was also upregulated in IgAN patients (Figure 2C,D, *p* < .05). Measurements of urinary FUT8 activity using HLCP indicated no difference in these two groups (*p* > .05, Figure S1).

### 3.3 IgAN patients had upregulation of renal FUT8 protein and a positive correlation of renal FUT8 protein with serum FUT8 activity

**Figure 3:**
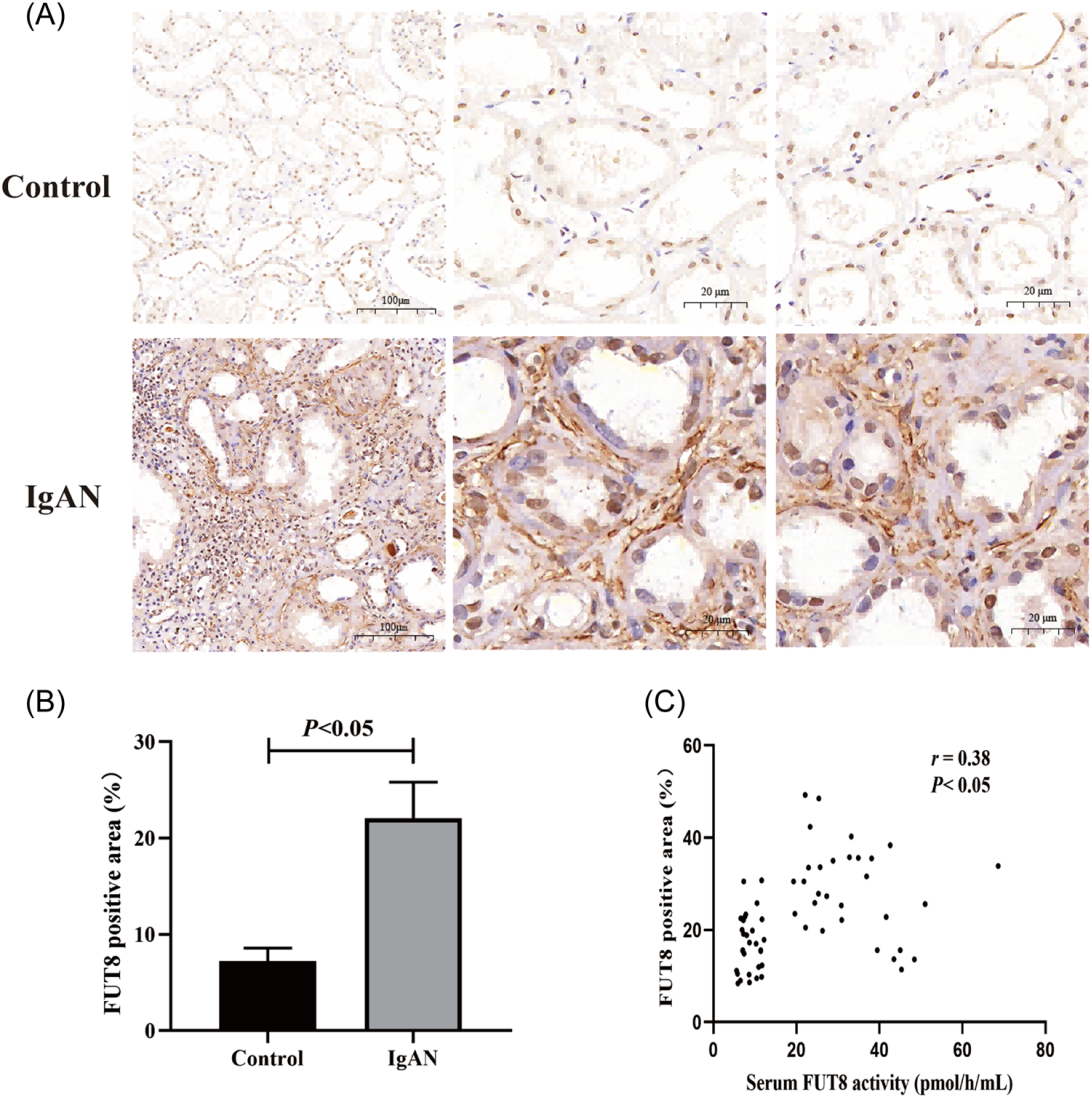
IgAN patients had upregulation of FUT8 protein in renal tissues and a positive correlation of renal FUT8 protein with serum FUT8 activity. (A) Representative immunohistochemical images of FUT8 protein in renal sections of a control patient and IgAN patient (×100/400). (B) Quantitative analysis of FUT8 protein expression in all controls and IgAN patients. (C) Spearman correlation analysis of serum FUT8 activity and FUT8 protein expression in renal sections of IgAN patients. FUT8, α‐1,6 fucosyltransferase; IgAN, immunoglobulin A nephropathy

We also measured FUT8 protein expression in the renal tissues using immunohistochemistry. The results indicated that expression of FUT8 protein was significantly higher in the renal tubulointerstitium of IgAN patients than healthy controls (Figure 3A,B). Moreover, serum FUT8 activity positively correlated with the renal expression of FUT8 protein in IgAN patients (*r* = .38, *p* < .05, Figure 3C).

### 3.4 Serum FUT8 activity was independently associated with serum creatinine and eGFR

We then analyzed the association of serum FUT8 activity with different clinical parameters in IgAN patients. Spearman correlation analysis indicated that serum FUT8 activity was positively correlated with serum creatinine (*r* = .330, *p* < .05) and urinary protein (*r* = .191, *p* < .05), and negatively correlated with eGFR (*r* = −0.206, *p* < .05; Supplementary Figure 2). Multivariable logistic regression analyses showed that serum FUT8 activity was significantly associated with serum creatinine (odds ratio [OR] = 2.07, 95% confidence interval [CI] [1.42–3.03]) and eGFR (OR = 0.60, 95% CI [0.44–0.82]) in crude model. Similar results were found after adjusting for all covariates in Table 2. The results demonstrated that serum FUT8 activity was independently associated with serum creatinine and eGFR.

**Table 2:** Multivariable logistic regression analyses for serum FUT8, serum creatinine, and eGFR

Item	Crude model (95%CI)	*p*	Model I (95%CI)	*p*	Model II(95%CI)	*p*
Scr	2.07 [1.42–3.03]	<.001	2.07 [1.42–3.02]	<.001	1.92 [1.55–2.73]	<.001
eGFR	0.60 [0.44–0.82]	<.001	0.59 [0.43–0.80]	<.001	0.60 [0.47–0.77]	<.001

*Note*: Crude model: unadjusted. Adjust model I: adjusted for age, sex. Adjust model II: adjusted for age, sex, mean arterial pressure, albumin, hemoglobin, cholesterol, blood urea nitrogen, and uric acid.

Abbreviations: CI, confidence interval; FUT8, α‐1,6 fucosyltransferase; eGFR, estimated glomerular filtration rate; Scr, serum creatinine.

### 3.5 IgAN patients with high serum FUT8 activity had more severe interstitial injury and worse renal function

**Figure 4:**
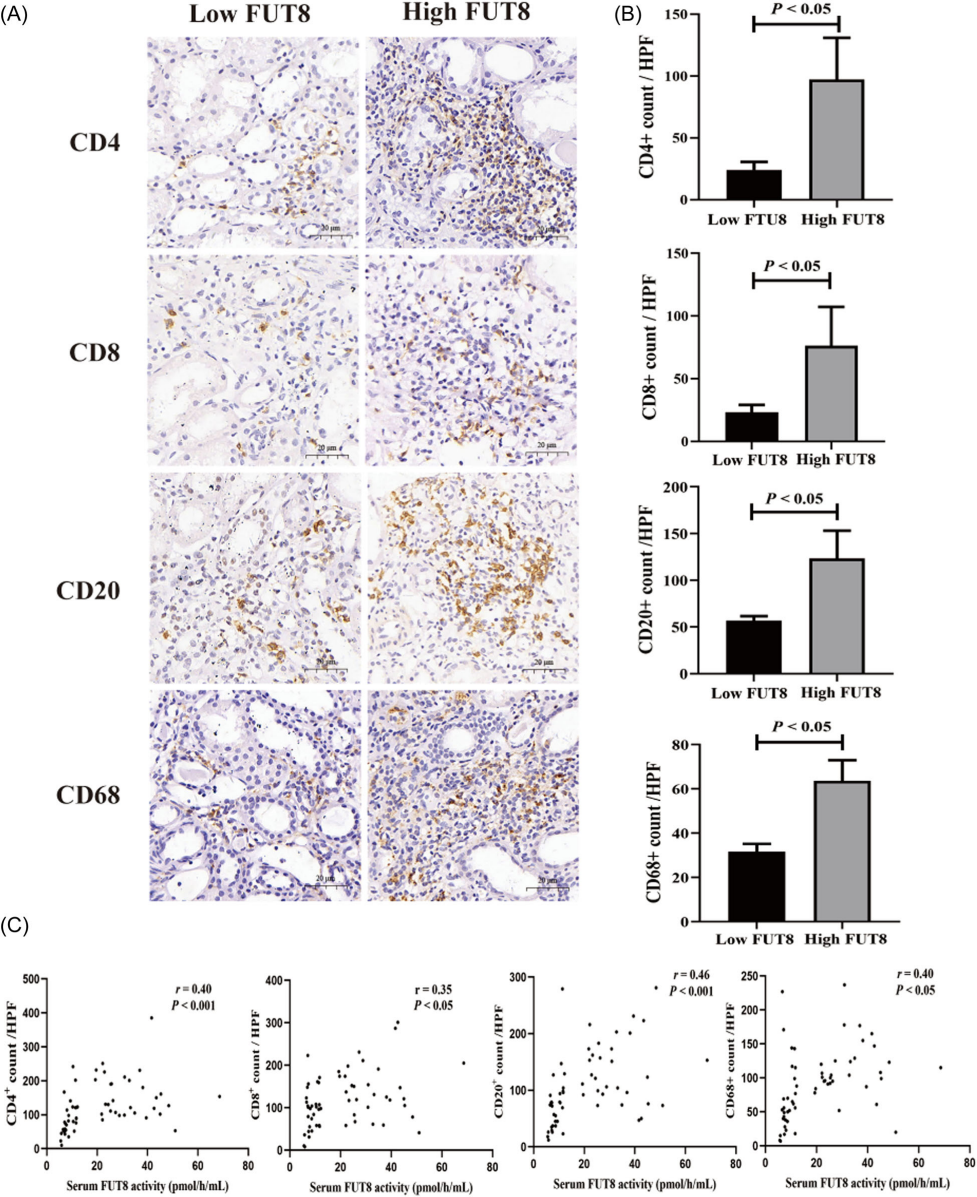
IgAN patients with high serum FUT8 activity had more inflammatory cells infiltration. (A) Representative immunohistochemical images of CD4^+^ T cells, CD8^+^ T cells, CD20^+^ B cells, and CD68^+^ macrophages in IgAN patients with low and high serum FUT8 activity (×400). (B) Quantitative analysis of the infiltration of CD4^+^ T cells, CD8^+^ T cells, CD20^+^ B cells, and CD68^+^ macrophages in all IgAN patients with low and high serum FUT8 activity. (C) Spearman correlation analysis of serum FUT8 activity with the counts of renal infiltration by CD4^+^ T cells, CD8^+^ T cells, CD20^+^ B cells, and CD68^+^ macrophages. FUT8, α‐1,6 fucosyltransferase

**Figure 5:**
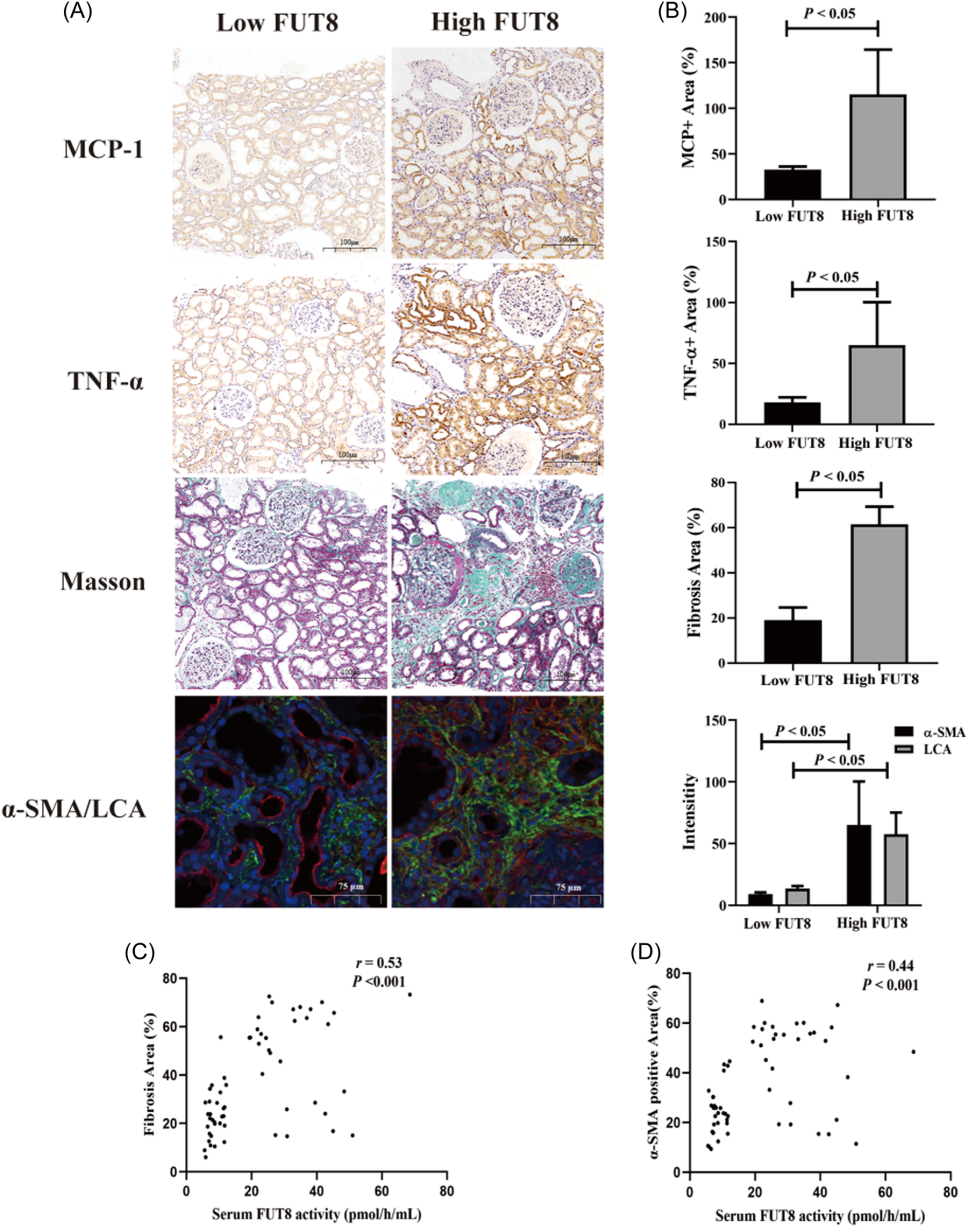
IgAN patients with high serum FUT8 activity had more pro‐inflammatory cytokines and fibrosis marker in renal tissues. (A) Representative immunohistochemical images of MCP‐1 and TNF‐α (×100), Masson staining (×100), and immunofluorescence staining for α‐SMA and LCA (×200) in IgAN patients with low and high serum FUT8 activity. (B) Quantitative analysis of MCP‐1, TNF‐α, fibrosis area, α‐SMA, and LCA in all IgAN patients with low and high serum FUT8 activity. (C) Spearman correlation analysis of serum FUT8 activity with fibrosis area in renal sections. (D) Spearman correlation analysis of serum FUT8 activity with α‐SMA‐positive area in renal sections. FUT8, α‐1,6 fucosyltransferase; IgAN, immunoglobulin A nephropathy; TNF‐α, tumor necrosis factor‐α

To compare the pathology of IgAN patients with different serum FUT8 activity, we divided these patients into a low serum FUT8 activity group (≤12.2 pmol/h/ml, *n* = 40) and a high serum FUT8 activity group (>12.2 pmol/h/ml, *n* = 95) using a cut‐off value determined from ROC curve analysis (Figure S3). We found that IgAN patients with high serum FUT8 activity had a higher T score in the Oxford classification and interstitial inflammation index (both *p* < .05, Supplementary Table). The immunohistochemistry data showed that patients in the high serum FUT8 activity group had more CD4^+^ T cells, CD8^+^ T cells, CD20^+^ B cells, and CD68^+^ macrophages in their renal tissues (all *p* < .05, Figure 4A,B). Moreover, patients with high serum FUT8 activity had increased expression of MCP‐1 and TNF‐α, greater fibrotic area, and a higher α‐SMA/LCA ratio (all *p* < .05, Figure 5A,B). We further investigated the relationship between serum FUT8 activity and infiltration of inflammatory cells and fibrosis. The results indicated that serum FUT8 activity had positive correlations with the counts of CD4^+^ T cells, CD8^+^ T cells, CD20^+^ B cells, and CD68^+^ macrophages (all *p* < .05, Figure 4C), and with fibrotic area and the level of α‐SMA (*p* < .05, Figure 5C,D).

Patients with high serum FUT8 activity had a higher serum creatinine (90.6 vs. 75.2 μmol/L, *p* < .05) and a lower eGFR (85.7 vs. 101.1 ml/min/1.73 m^2^, *p* < .05, Table 3). This indicated that IgAN patients with high serum FUT8 activity had worse renal function than those with low serum FUT8 activity.

**Table 3:** Clinical characteristics of IgAN patients with low and high serum FUT8 activity

Item	Low serum FUT8 activity	High serum FUT8 activity	*p*
	(≤12.2 pmol/h/ml)	(>12.2 pmol/h/ml)	
	*n* = 40	*n* = 95	
Male [*n* (%)]	18 (45%)	36 (38%)	NS
Age (years)	35 (28, 45)	36 (30, 49)	NS
Mean arterial pressure (mmHg)	105 (100, 110)	105 (97, 115)	NS
Albumin (g/L)	39.7 (35.6, 43.0)	38.9 (36.3, 41.9)	NS
Cholesterol (mmol/L)	4.9 (4.3, 5.6)	4.9 (4.2, 5.7)	NS
Hemoglobin (g/L)	136.3 ± 15.9	131.0 ± 18.3	NS
eGFR (ml/min/1.73 m^2^)	101.0 (91.8, 115.8)	85.7 (66.9, 104.7)	**<.05**
Serum creatinine (μmol/L)	75.2 (56.3, 83.3)	90.6 (63, 107)	**<.05**
Blood urea nitrogen (mmol/L)	6.0 (4.6, 7.6)	5.7 (4.7, 7.2)	NS
Uric acid (mmol/L)	368.8 ± 100.5	385.7 ± 104.0	NS
Urine protein (mg/24 h)	831.5 (326.5, 1712.8)	1215.0 (573.0, 2295.0)	NS
Microscopic hematuria [*n* (%)]	36 (90.0%)	89(93.7%)	NS

*Note*: Values are expressed as means ± standard deviations or median (25th, 75th percentiles).

Abbreviation: eGFR, estimated glomerular filtration rate; FUT8, α‐1,6 fucosyltransferase; IgAN, immunoglobulin A nephropathy; NS, no significance.

## 4 DISCUSSION

In the present study, we found that serum FUT8 activity and serum FUT8 protein were elevated in IgAN patients. Serum FUT8 activity also had a positive correlation with expression of FUT8 in the renal tissues of IgAN patients. Serum FUT8 activity was independently associated with serum creatinine and eGFR. The high serum FUT8 activity group (>12.2 pmol/h/ml) had more inflammation cell infiltration and severe interstitial fibrosis, worse renal function than the low serum FUT8 activity group. Collectively Our study for the first time found that serum FUT8 activity, FUT8 protein, and renal FUT8 protein expression were upregulated, and serum FUT8 enzyme activity was significantly correlated with renal interstitial injury in IgAN patients. This study for the first time showed the relationship between FUT8, a key enzyme in the process of core fucosylation, and renal tubulointerstitial lesion in IgAN nephropathy. Serum FUT8 activity may be an ideal biomarker for evaluating renal interstitial damage in IgAN patients.

A kidney biopsy remains the only way to evaluate renal interstitial injury in IgAN. While kidney biopsy is invasive and can not monitor the real‐time renal interstitial damage change during progression of IgAN. It is necessary to search for noninvasive biomarker to evaluate renal interstitial lesion in IgAN. Our previous research found that blockade of FUT8 had protective effects on the renal tubular cell epithelial‐mesenchymal transition in vitro and renal fibrosis in vivo.^14^, ^15^, ^16^ It is suggested that FUT8 may be an important hub in renal interstitial fibrosis. However, it remained unknown whether FUT8 also contributed to renal interstitial damage in IgAN. The results presented here are the first to indicate that serum FUT8 activity and serum FUT8 protein were significantly increased in IgAN patients. We also found that the expression of FUT8 protein was upregulated in the renal tissues in IgAN patients, and that this was positively correlated with serum FUT8 activity. Thus, our results suggested that upregulation of FUT8 in the systemic circulation and in renal tissues maybe a characteristic feature of IgAN and was the first investigation to examine the role of FUT8 in IgAN.

As we all known, aberrant O‐glycosylation of degalactosylated IgA (gd‐IgA) is involved in pathogenesis in IgAN.^17^, ^18^ while there is little known about the abnormal *N*‐linked glycans of IgA in IgAN patients. Dotz recently reported fucosylation of the *N*‐glycopeptides is upregulated in IgAN patients.^19^ However, the relationship of core fucosylation of the *N*‐linked glycans and IgAN is unclear. Our present study first found that serum FUT8 activity was upregulated in IgAN patients and was significantly associated with tubulointerstitial injury in IgAN patients. This finding maybe identified FUT8 as a participant in the pathogenesis of IgAN, These results provided an important basis for *N*‐linked glycans in the pathogenesis of IgAN.

Previous studies suggested that proteinuria and reduced renal function at the time of renal biopsy were associated with poor renal outcome in IgAN patients.^3^ We therefore further examined the relationships of serum FUT8 activity, proteinuria, and renal function in IgAN patients. Multivariable logistic regression analyses adjusted for a variety of clinical parameters showed that serum FUT8 activity was independently correlated with serum creatinine and eGFR at the time of renal biopsy. Also, our study demonstrated that IgAN patients with high serum FUT8 activity had worse renal function than that of low serum FUT8 activity. Therefore, serum FUT8 activity has potential approach as an early predictor of renal function deterioration in IgAN patients. However, the association between serum FUT8 activity and renal outcome remains unclear. A further study with a longer follow‐up duration to examine their relationship is needed.

A variety of immune cells and inflammatory cells, such as T lymphocyte subsets, B lymphocytes, and macrophages, function in the pathogenesis of IgAN.^20^ The infiltration of inflammatory cells is an early and prominent histopathologic change during IgAN, and this precedes tubular atrophy and interstitial fibrosis.^21^ However, there is no validated means, even the Oxford classification, for assessments of renal interstitial infiltration of inflammatory cells in IgAN. Previous researchers reported that FUT8‐mediated core fucosylation plays a significant role in the activation of T cells, B cells, and macrophages.^12^, ^22^, ^23^ Our findings demonstrated that more CD4^+^ T cells, CD8^+^ T cells, CD20^+^ B cells, and CD68^+^ macrophages were present in the renal tissues of IgAN patients with high serum FUT8 activity, and that serum FUT8 activity had a significantly positive association with the counts of these inflammatory cells in renal tissues. Our results suggested that serum FUT8 activity was associated with inflammatory cell infiltration in renal tissues in IgAN patients. It is first reported the relationship of FUT8 participated and inflammatory response in IgAN. In addition, our results suggested that FUT8 may, at least in part, mediate renal injury by promoting the infiltration of inflammatory cells in IgAN, which also provides a new mechanism for FUT8 to participate in renal interstitial injury in IgAN.

Furthermore, we also analyzed the correlation between serum FUT8 activity and renal pathological injury. Our results showed that IgAN patients with high serum FUT8 activity had a higher T score and interstitial inflammation index than that of low serum FUT8 activity. Serum FUT8 activity was also correlated to some inflammation cell infiltration and fibrosis markers, confirming that serum FUT8 activity was related to renal inflammation and interstitial fibrosis, and suggesting that FUT8 may function in the renal interstitial injury in IgAN. The T‐score of Oxford classification requires pathological evaluation of renal biopsy and cannot dynamically monitor IgAN tubulointerstitial injury. Serum FUT8 activity is related to IgAN tubulointerstitial injury, which may become an ideal noninvasive biological marker for the evaluation of IgAN tubulointerstitial injury in the future. As mentioned previously, FUT8 appears to play a central role in the activation of multiple inflammatory cells.^12^, ^22^, ^23^ Based on our previous in vivo findings, there is increased activation of multiple signaling pathways, such as TGF‐β/Smad and PDGF/ERK, during renal interstitial and peritoneal fibrosis, and inactivation of these pathways by knockdown of FUT8 significantly attenuated fibrotic processes.^14^, ^15^, ^24^, ^25^ This suggests that FUT8 is essential for the development of inflammation and fibrosis. Activation of inflammatory cascades and multiple fibrogenic signaling pathways are well known to induce the pathogenesis and progression of renal interstitial injury in IgAN.^26^, ^27^, ^28^ Our findings are the first to demonstrate that serum FUT8 activity was associated with renal interstitial inflammation and fibrosis in IgAN. Our results thus suggest that blockade of FUT8 provide a novel therapeutic strategy for IgAN by downregulation of multiple inflammatory cascades and fibrogenic signaling pathways.

There are several limitations in the present study. First, although we identified increased serum FUT8 activity in IgAN patients, the mechanism is unclear. Second, serum FUT8 activity and FUT8 protein in other renal disease need to further confirmed. Third, we only assessed serum FUT8 activity at baseline, and did not monitor serum FUT8 activity change during follow‐up. The relationship of serum FUT8 activity and IgAN progression was unclear.

## 5 CONCLUSION

In conclusion, serum FUT8 activity was positive association with renal tubulointerstitial injury in IgAN patients and suggested that serum FUT8 activity has potential for use as a noninvasive biomarker to evaluate tubulointerstitial injury in IgAN patients. Moreover, blockade of FUT8 maybe an effective therapeutic approach for the future treatment of IgAN patients.

## AUTHORS CONTRIBUTIONS

Ning Yang contributed to analyze data and writing the manuscript. Hong‐li Lin was responsible for the study design, supervised data collection, and edit the manuscript. Hui He, Xia‐nan Guo, and Xue‐feng Yuan contributed to measure serum and urinary FUT8 activity. Long‐kai Li contributed to collect and summarize clinical data, and perform statistical analyses. Zhi‐tong Li contributed to follow‐up. Wei‐dong Wang, Biao‐jie Qin, and Xiang‐ning Du contributed to prepare and stain of renal pathological sections. Xu Zhang and Shu‐ni Chen contributed to assess renal pathology.

## CONFLICTS OF INTEREST

The authors declare no conflicts of interest.

## Supporting information


**Supplement Figure 1. Urine FUT8 activity in controls and IgAN patients** (A) Representative HPLC images. (B) Quantitation of results.

**Supplement Figure 2. Spearman correlation of serum FUT8 activity with SCr (A), urine protein (B), eGFR (C), and MAP (D)**. eGFR, estimated glomerular filtration rate; MAP, mean arterial pressure; Scr, serum creatinine.

**Supplement Figure 3. Receiver operating characteristic curve based on serum FUT8 activity**. Optimal cut‐off: 12.2 pmol/h/mL.

**Supplement Table. The differences in renal pathology of IgAN patients with low and high serum FUT8 activity**.

## References

[1] Rodrigues JC , Haas M , Reich HN . IgA nephropathy. Clin J Am Soc Nephrol. 2017;12:677‐686. 10.2215/cjn.07420716 28159829 PMC5383386

[2] Rychlik I , et al. Clinical features and natural history of IgA nephropathy. Ann Med Interne. 1999;150:117‐126. 10392260

[3] Barbour SJ , Espino‐Hernandez G , Reich HN , et al. The MEST score provides earlier risk prediction in lgA nephropathy. Kidney Int. 2016;89:167‐175. 10.1038/ki.2015.322 26759049

[4] Falk MC , Ng G , Zhang GY , et al. Infiltration of the kidney by alpha beta and gamma delta T cells: effect on progression in IgA nephropathy. Kidney Int. 1995;47:177‐185. 10.1038/ki.1995.21 7731144

[5] Silva GEB , Costa RS , Ravinal RC , et al. Renal macrophage infiltration is associated with a poor outcome in IgA nephropathy. Clinics. 2012;67:697‐703. 10.6061/clinics/2012(07)01 22892911 PMC3400157

[6] Takahashi M , Kuroki Y , Ohtsubo K , Taniguchi N . Core fucose and bisecting GlcNAc, the direct modifiers of the N‐glycan core: their functions and target proteins. Carbohydr Res. 2009;344:1387‐1390. 10.1016/j.carres.2009.04.031 19508951

[7] Yang Q , Wang LX . Mammalian α‐1,6‐Fucosyltransferase (FUT8) is the sole enzyme responsible for the N‐acetylglucosaminyltransferase I‐independent core fucosylation of high‐mannose N‐glycans. J Biol Chem. 2016;291:11064‐11071. 10.1074/jbc.M116.720789 27008861 PMC4900256

[8] Calderon AD , et al. Substrate specificity of FUT8 and chemoenzymatic synthesis of core‐fucosylated asymmetric N‐glycans. Org Biomol Chem. 2016;14:4027‐4031. 10.1039/c6ob00586a 27080952 PMC4852481

[9] Trimarchi H , Barratt J , Cattran DC , et al. Oxford classification of IgA nephropathy 2016: an update from the IgA nephropathy classification working group. Kidney Int. 2017;91:1014‐1021. 10.1016/j.kint.2017.02.003 28341274

[10] Jiang L , LIU G , LV J , et al. Concise semiquantitative histological scoring system for immunoglobulin A nephropathy. Nephrology (Carlton). 2009;14:597‐605. 10.1111/j.1440-1797.2008.01083.x 19422527

[11] Luo R , Guo SM , Li YQ , et al. Plasma fractalkine levels are associated with renal inflammation and outcomes in immunoglobulin A nephropathy. Nephrol, Dialysis, Transplant. 2019;34:1549‐1558. 10.1093/ndt/gfy169 30010903

[12] Liang W , Mao S , Sun S , et al. Core fucosylation of the T cell receptor is required for T cell activation. Front Immunol. 2018;9:78. 10.3389/fimmu.2018.00078 29434598 PMC5796888

[13] Ihara H , Tsukamoto H , Taniguchi N , Ikeda Y . An assay for α 1,6‐fucosyltransferase (FUT8) activity based on the HPLC separation of a reaction product with fluorescence detection. Methods Mol Biol. 2013;1022:335‐348. 10.1007/978-1-62703-465-4_25 23765673

[14] Wang N , Deng Y , Liu A , et al. Novel mechanism of the pericyte‐myofibroblast transition in renal interstitial fibrosis: core fucosylation regulation. Sci Rep. 2017;7:16914. 10.1038/s41598-017-17193-5 29209018 PMC5717002

[15] Shen N , Lin H , Wu T , et al. Inhibition of TGF‐β1‐receptor posttranslational core fucosylation attenuates rat renal interstitial fibrosis. Kidney Int. 2013;84:64‐77. 10.1038/ki.2013.82 23486519

[16] Lin H , Wang D , Wu T , et al. Blocking core fucosylation of TGF‐β1 receptors downregulates their functions and attenuates the epithelial‐mesenchymal transition of renal tubular cells. Am J Physiol Renal Physiol. 2011;300:F1017‐F1025. 10.1152/ajprenal.00426.2010 21228108

[17] Perše M , Večerić‐Haler Ž . The role of IgA in the pathogenesis of IgA nephropathy. Int J Mol Sci. 2019;20:6199. 10.3390/ijms20246199 PMC6940854 31818032

[18] Floege J , Barbour SJ , Cattran DC , et al. Management and treatment of glomerular diseases (part 1): conclusions from a kidney disease: improving global outcomes (KDIGO) controversies conference. Kidney Int. 2019;95:268‐280. 10.1016/j.kint.2018.10.018 30665568

[19] Dotz V , Visconti A , Lomax‐Browne HJ , et al. O‐ and N‐Glycosylation of serum immunoglobulin A is associated with IgA nephropathy and glomerular function. J Am Soc Nephrol. 2021;32:2455‐2465. 10.1681/asn.2020081208 34127537 PMC8722783

[20] Chang S , Li XK . The role of immune modulation in pathogenesis of IgA nephropathy. Front Med. 2020;7:92. 10.3389/fmed.2020.00092 PMC7105732 32266276

[21] Lai KN , Chan LY , Leung JC . Mechanisms of tubulointerstitial injury in IgA nephropathy. Kidney Int Suppl . 2005:S110‐S115. 10.1111/j.1523-1755.2005.09426.x 15752226

[22] Nakayama K , Wakamatsu K , Fujii H , et al. Core fucose is essential glycosylation for CD14‐dependent toll‐like receptor 4 and toll‐like receptor 2 signalling in macrophages. J Biochem. 2019;165:227‐237. 10.1093/jb/mvy098 30445455

[23] Li W , Yu R , Ma B , et al. Core fucosylation of IgG B cell receptor is required for antigen recognition and antibody production. J Immunol. 2015;194:2596‐2606. 10.4049/jimmunol.1402678 25694612

[24] Li L , Shen N , Wang N , et al. Inhibiting core fucosylation attenuates glucose‐induced peritoneal fibrosis in rats. Kidney Int. 2018;93:1384‐1396. 10.1016/j.kint.2017.12.023 29571940

[25] Yu C , Yang N , Wang W , et al. Blocking core fucosylation of epidermal growth factor (EGF) receptor prevents peritoneal fibrosis progression. Ren Fail. 2021;43:869‐877. 10.1080/0886022x.2021.1918557 33993842 PMC8143636

[26] Sheng X , Zuo X , Liu X , Zhou Y , Sun X . Crosstalk between TLR4 and Notch1 signaling in the IgA nephropathy during inflammatory response. Int Urol Nephrol. 2018;50:779‐785. 10.1007/s11255-017-1760-2 29230705

[27] Bao H , Hu S , Zhang C , et al. Inhibition of miRNA‐21 prevents fibrogenic activation in podocytes and tubular cells in IgA nephropathy. Biochem Biophys Res Commun. 2014;444:455‐460. 10.1016/j.bbrc.2014.01.065 24468088

[28] Wu MY , Chen CS , Yiang GT , et al. The emerging role of pathogenesis of IgA nephropathy. J Clin Med . 2018;7. 10.3390/jcm7080225 PMC6112037 30127305

