# Supplementary figures and images for: Positive association of serum FUT8 activity with renal tubulointerstitial injury in IgA nephropathy patients
Source: Immun Inflamm Dis. 2022 Aug 29;10(9):e686. doi: 10.1002/iid3.686 (PMC9425009; doi:10.1002/iid3.686)

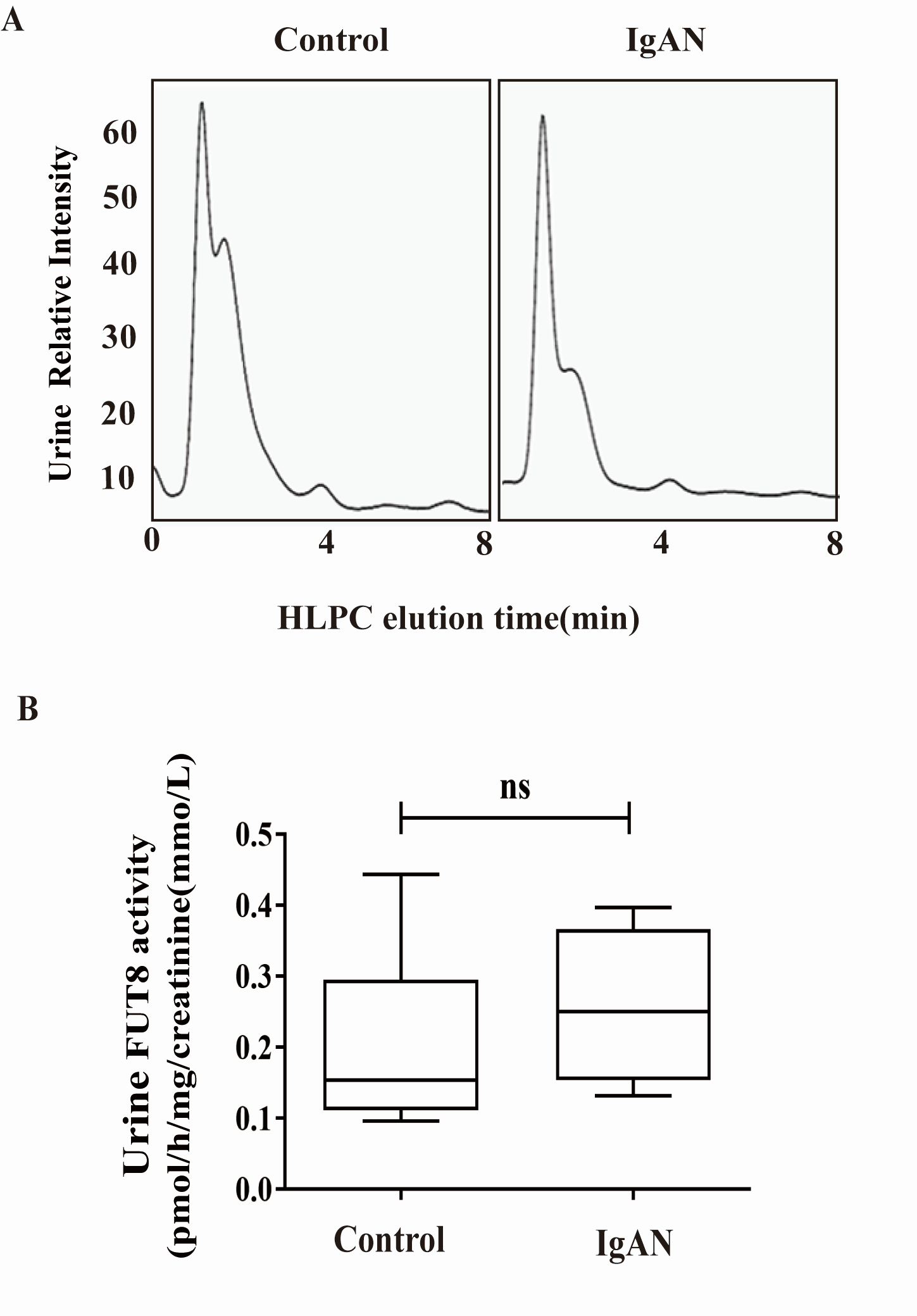

Supplement: Supplementary file 1 — Supplement Figure 1. Urine FUT8 activity in controls and IgAN patients (A) Representative HPLC images. (B) Quantitation of results. [file IID3-10-e686-s004.png]

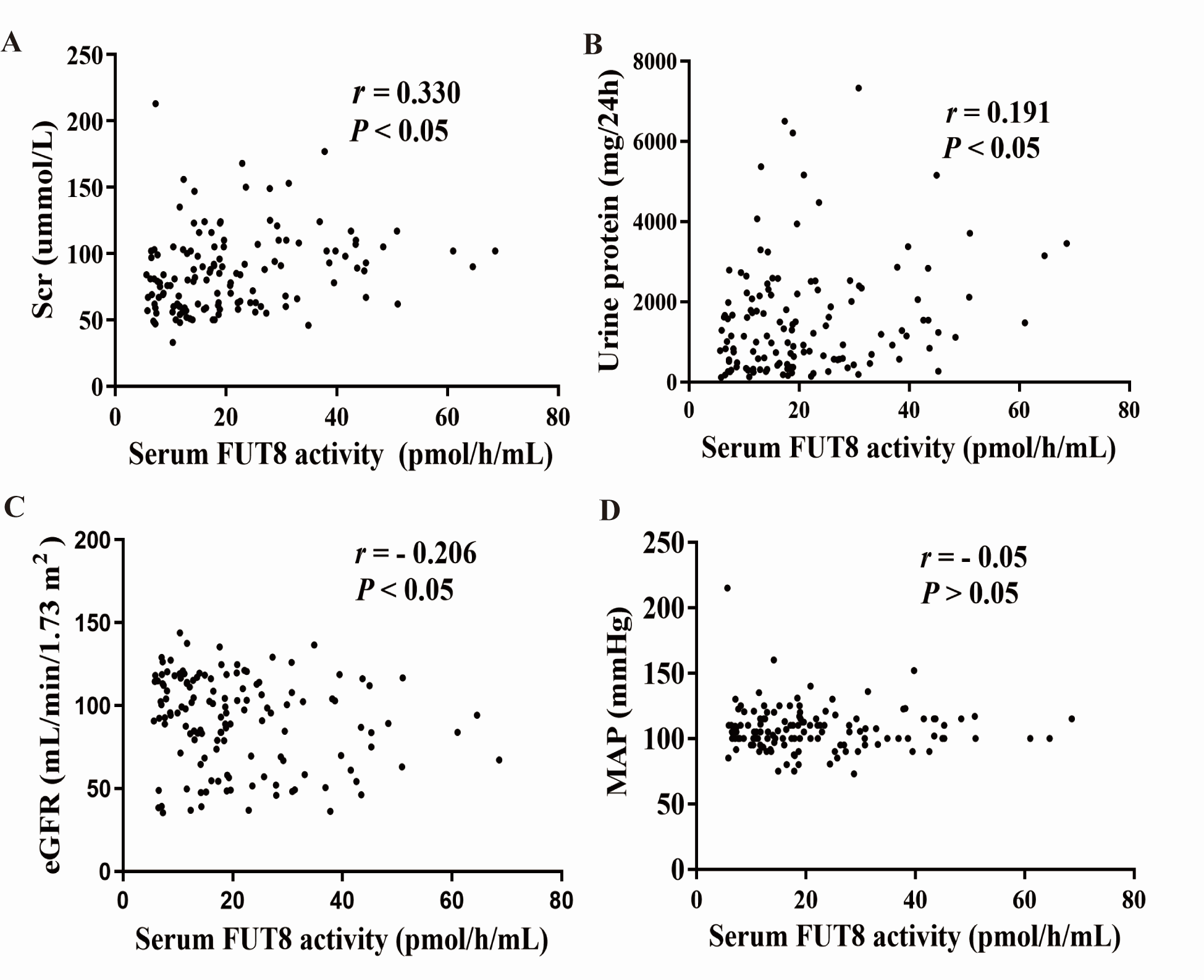

Supplement: Supplementary file 2 — Supplement Figure 2. Spearman correlation of serum FUT8 activity with SCr (A), urine protein (B), eGFR (C), and MAP (D). eGFR, estimated glomerular filtration rate; MAP, mean arterial pressure; Scr, serum creatinine. [file IID3-10-e686-s003.png]

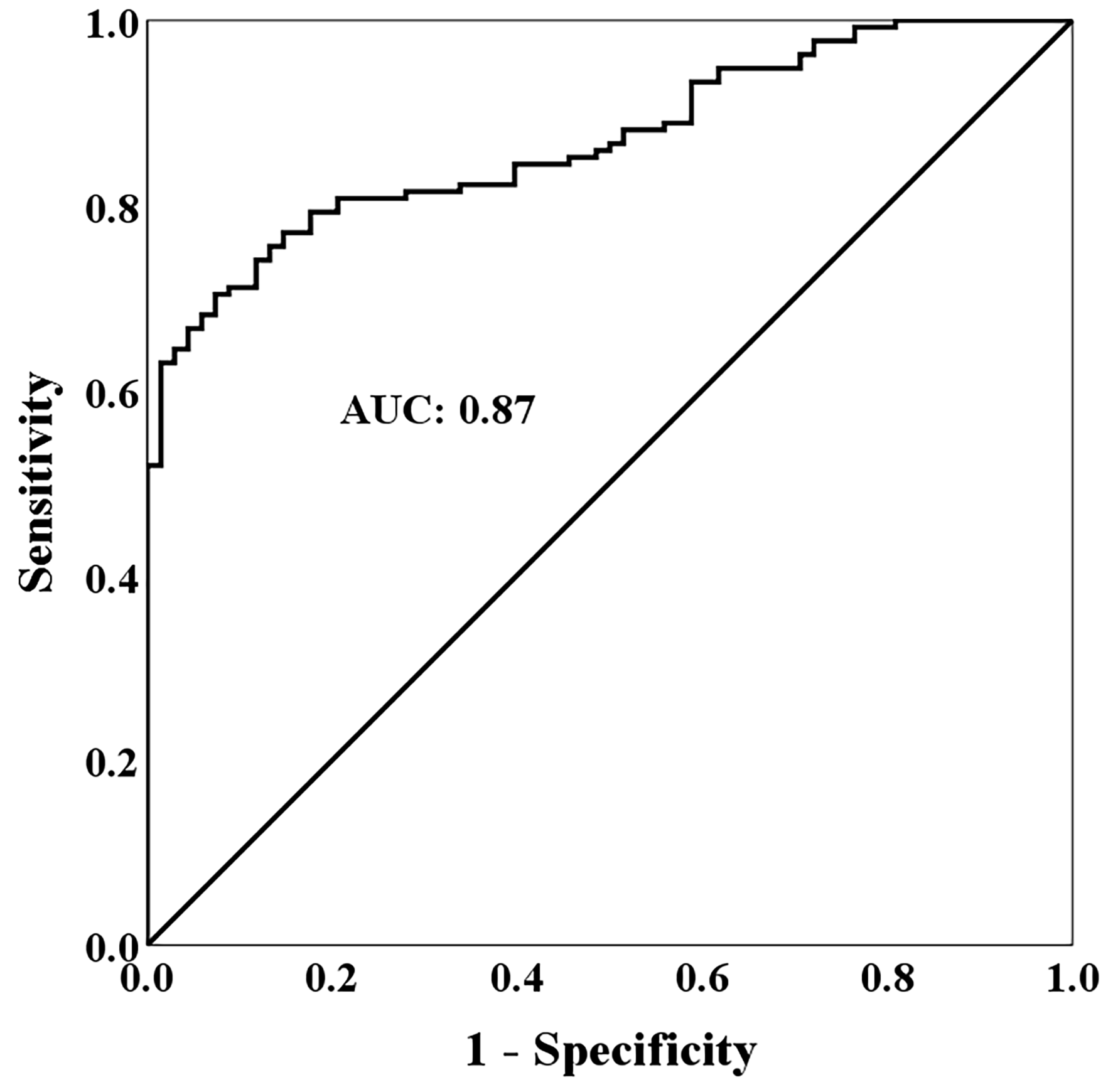

Supplement: Supplementary file 3 — Supplement Figure 3. Receiver operating characteristic curve based on serum FUT8 activity. Optimal cut‐off: 12.2 pmol/h/mL. [file IID3-10-e686-s002.tif]
