# Supplementary material for: Positive association of serum FUT8 activity with renal tubulointerstitial injury in IgA nephropathy patients
Source: Immun Inflamm Dis. 2022 Aug 29;10(9):e686. doi: 10.1002/iid3.686 (PMC9425009; doi:10.1002/iid3.686)
Supplement: Supplementary file 4 — Supplement Table. The differences in renal pathology of IgAN patients with low and high serum FUT8 activity. [file IID3-10-e686-s001.docx]

**Supplement Table. The differences in renal pathology of IgAN patients with low and high serum FUT8 activity**

|  | **Low serum FUT8 activity** | **High serum FUT8 activity** | **χ^2^** | ***P*** |
| --- | --- | --- | --- | --- |
|  | (≤12.2 pmol/h/mL) | (>12.2 pmol/h/mL) |  |  |
| **M** |  |  |  |  |
| 0 | 22.5% | 23.2% | 0.468 | 1 |
| 1 | 77.5% | 76.8% | — | — |
| **E** |  |  |  |  |
| 0 | 100.0% | 97.9% | 0.21 | 0.885 |
| 1 | 0.0% | 2.1% | — | — |
| **S** |  |  |  |  |
| 0 | 35.0% | 23.2% | 1.458 | 0.227 |
| 1 | 65.0% | 76.8% | — | — |
| **T** |  |  |  |  |
| 0 | 42.5% | 27.4% | 10.081 | **0.016** |
| 1 | 22.5% | 36.8% | — | — |
| 2 | 35.0% | 24.2% | — | — |
| 3 | 0.0% | 11.6% | — | — |
| C |  |  |  |  |
| 0 | 97.5% | 96.8% | 0 | 1 |
| 1 | 2.5% | 3.2% | — | — |
| **Inf** |  |  |  |  |
| 0 | 57.5% | 14.7% | 29.449 | **0** |
| 1 | 30.0% | 31.6% | — | — |
| 2 | 10.0% | 42.1% | — | — |
| 3 | 2.5% | 11.6% | — | — |
| **VCI** |  |  |  |  |
| 0 | 0.0% | 3.2% | — | — |
| 1 | 20.0% | 13.7% | 3.543 | 0.456 |
| 2 | 37.5% | 44.2% |  |  |
| 3 | 32.5% | 34.7% | — | — |
| 4 | 10.0% | 4.2% | — | — |

Note: M, mesangial hypercellularity; E, endocapillary hypercellularity; S, segmental sclerosis; T, interstitial fibrosis/tubular atrophy; C, crescents; Inf, interstitial inflammation index; VCI, vascular chronic index.
